# Comparative Expenditure of Various Hospitals for the Year 1886

**Published:** 1887-12-31

**Authors:** 


### COMPARATIVE EXPENDITURE OF VARIOUS HOSPITALS FOR THE YEAR 1886,

**With the Average Cost for each Patient and Occupied Bed also, detailed under Nine Heads.**

**COMPILED FROM THE RESPECTIVE REPORTS BY THOMAS PARKINSON, BURY.**

| Out-Patients No.* | Out-Patients Cost per Patient. | NAME OF HOSPITAL. | No. of In-Patients. | Average No. of Beds Occupied. | No. of Resident Officials. | Average Cost per Patient per Week | Average Cost per Occupied Bed. | Provisions. | §Stimulants. | ‖Domestic Expenses. | ¶Surgery and Dispensary Expenses. | **Salaries and Wages. | ††Management Expenses. | ‡‡Rates and Insurance. | Repairs and Alterations. | Garden and Miscellaneous Expenses. |
|---|---|---|---|---|---|---|---|---|---|---|---|---|---|---|---|---|
| None | .. | Keighley Cottage | 86 | ... | 3 | 0 15 4 | 62 7 11 | 18 10 4 | Included in Provisions | ... | ... | 14 11 6 | ... | ... | ... | ... |
| 3903 | 1 9⅝ | Rotherham | 189 | 17·8 | 13 | 1 2 3⅞ | 64 10 6 | 27 12 10¼ | 1 4 5¾ | 15 6 1¼ | 0 10 0¾ | 15 5 4½ | 0 4 0½ | 0 3 4½ | 2 10 7¼ | 1 13 7¼ |
| 1909 | 4 0 $\frac{9}{20}$ | Rochdale | 196 | 18·40 | 7 | 0 19 7¼ | 51 7 3 | 20 13 0 | 2 2 7½ | 9 16 0½ | 1 7 6 | 14 7 11½ | 0 5 8 | 1 17 3½ | 0 17 2 | ... |
| 3061 | 1 8 | Doncaster | 212 | 17·42 | 12 | 1 6 0 13/[illegible] | †68 6 4 | 32 5 9 | 1 9 7½ | 12 1 11½ | 0 10 7 | 14 12 6 | 0 2 4 | 0 18 3½ | 4 9 5½ | 1 15 9½ |
| 1851 | 3 7½ | Chesterfield and North Derbyshire | 240 | 26·4 | 10 | 0 16 0¼ | 43 5 11 | 15 19 4 | 0 18 10 | 6 3 5½ | 0 9 8½ | 9 7 8½ | 1 14 4½ | 0 10 4½ | 7 6 11½ | 0 15 2 |
| 1699 | 5 7½ | Dewsbury and District | 283 | 33·0 | 11 | 0 16 0½ | 43 8 7¼ | 17 10 5 | 0 12 3 | 8 19 8 | 1 13 7 | 9 0 0 | 1 16 3¼ | 0 8 6¼ | 2 17 10½ | 0 10 0 |
| 3377 | 3 11 $\frac{3}{16}$ | Wakefield (Clayton) | 318 | 31·0 | 14 | 1 3 10¾ | 62 18 5½ | 24 14 0 | 2 16 4¼ | 14 10 4 | 1 14 7½ | 12 11 2 | 0 5 9¼ | 0 19 9 | 3 17 5¼ | 1 9 0 |
| 706 | 10 3 | Dorset County | 318 | 33·4 | ... | 1 6 1⅜ | ‡68 0 4 | 27 16 9 | 1 4 9½ | 13 1 10 | 2 0 1 | 13 15 7 | 1 18 8½ | 1 3 6 | 6 19 1 | ... |
| 3537 | 3 6 $\frac{11}{16}$ | Stockport | 621 | 40·0 | 20 | 1 2 11 | 58 14 7¼ | 28 12 5 | 1 4 3 | 8 16 11 | 0 17 7¾ | 13 7 1 | 0 13 7 | 0 19 4 | 2 5 6 | 1 17 10 |
| 2428 | 5 6¼ | Oldham | 625 | 72·6 | 22 | 0 16 1 | 42 5 5½ | 14 17 5 | 1 0 3½ | 10 14 10½ | 1 8 1 | 8 1 0 | 0 19 10½ | 0 6 1 | 4 14 5 | 0 3 5 |
| 3441 | 3 7⅞ | Macclesfield | 648 | 40·0 | ... | 1 1 9 | 56 12 8 | 21 4 5 | 1 15 5 | 12 1 7 | 1 7 3 | 12 5 0 | 0 17 6½ | 0 6 8½ | 4 17 8 | 1 17 1 |
| 2521 | 6 1 $\frac{15}{16}$ | Hereford | 654 | 70·0 | 19 | 1 4 6 | 34 8 1 | 16 1 10¼ | 2 2 10¾ | 5 6 1¾ | 1 7 11¼ | 6 12 2¼ | 0 15 2 | 0 13 1¼ | 1 7 5½ | 0 1 4¼ |
| None | ... | Harrogate (1884–5) | 738 | 52·71 | ... | 0 15 0½ | 31 12 2 | 15 16 1 | 0 2 1¾ | 3 9 8½ | 1 7 8½ | 8 10 0 | 0 15 7½ | 0 10 2 | 0 18 9 | 0 1 11¾ |
| 3836 | 6 3 $\frac{11}{16}$ | Halifax | 767 | 70·2 | 30 | 0 18 11½ | 49 7 11⅛ | 19 7 5⅛ | 1 3 5⅛ | 8 9 5⅞ | 2 6 1¾ | 12 11 10⅝ | 0 7 7⅝ | 1 19 5¼ | 3 2 5¾ | ... |
| 3695 | 6 2 | Bolton | 804 | 77·0 | 38 | 0 16 4½ | 42 15 5 | 17 9 11 | 1 16 7 | 7 12 5 | 1 11 9 | 9 16 4 | 0 9 7 | 0 8 2 | 3 10 8 | ... |
| 5940 | 3 7⅝ | Wigan, Royal Albert Edward | 890 | 85·0 | 40 | 0 18 0⅞ | 39 3 11 | 17 12 8¼ | 2 2 2 | 5 14 8 | 1 1 7 | 9 18 11 | 0 3 4 | 0 3 9 | 1 13 10 | 0 12 8 |
| 5349 | 4 6⅛ | Preston | 1239 | 91¼ | 38 | 0 19 0½ | 49 14 1 | 20 3 8 | 1 6 5 | 8 3 4 | 2 3 9¼ | 11 16 11¼ | 0 8 4 | 0 17 9¼ | 2 15 7 | 1 18 3¼ |
| 5329 | 3 8 | Blackburn | 1249 | 75·28 | 32 | 1 4 11¾ | 57 5 9 | 22 15 8 | 2 11 10 | 10 4 1 | 2 0 2 | 14 13 3 | 0 19 11 | 0 2 6 | 2 13 10 | 1 3 6 |
| None | ... | N. C. Mauldeth | ... | 82·5 | ... | 0 15 10 | 43 4 9 | 15 14 2 | 0 6 6 | 8 18 3 | 1 5 11 | 9 5 4 | 1 14 5½ | 2 18 9 | 1 10 8 | 1 10 8 |
| .. | ... | Buxton | 2449 | 151·2 | ... | 0 15 3 | 37 14 2 | 19 5 4½ | Included in Provisions. | 4 7 8½ | 1 5 0 | 8 4 0 | 1 8 3¼ | 0 13 3 | 2 6 0¼ | 0 4 6 |
| ... | 4 7⅝ | Average Cost of the above | ... | ... | ... | 0 19 8½ | 50 7 2½ | 20 14 2⅛ | 1 6 0½ | 9 3 1 | 1 7 10⅛ | 11 8 8¼ | 0 16 10⅜ | 0 16 10⅛ | 3 3 11⅝ | 0 16 6¾ |
| 1078 | 6 5⅛ | Bury | 248 | 21·09 | 15 | 1 10 9⅛ | 82 13 0 | 30 2 1 | 1 9 10 | 17 0 4 | 2 3 5 | 21 17 7 | 0 15 9 | 2 3 10 | 5 3 6 | 1 16 8 |

* Casuals and Dental Patients are not included.

† The cost per Bed in this Hospital is given as £45 11s. 11d.

‡ Includes new Beds, &c., for enlargement.

§ Includes Wine, Spirits, Beer, Mineral Waters.
‖ Include Coals, Gas, Water, Soap, &c., Drapery, Bedding, Furniture.
¶ Proportionate amount of these is charged to Out-Patients.

** Proportion of Doctor's Salary charged to Out-Patients.
†† Proportionate amount of these is charged to Out-Patients.
‡‡ In some towns the Hospital is not rated, nor any charge made for water.

